# Atypical presentation of an atypical pneumonia: a case report

**DOI:** 10.1186/s13256-022-03320-y

**Published:** 2022-03-16

**Authors:** Alvin Oliver Payus, Clarita Clarence, Tiong Nee, Wan Nur Nafisah Wan Yahya

**Affiliations:** 1 Medicine Based Department, Faculty of Medicine and Health Science, Universiti Malaysia Sabah (UMS), Jalan UMS, 88400 Kota Kinabalu, Sabah Malaysia; 2 Department of Internal Medicine, Universiti Kebangsaan Malaysia Medical Centre (UKMMC), Jalan Yaacob Latif, Cheras, 56000 Kuala Lumpur, Malaysia; 3 Hospital Rehabilitasi Cheras, Jalan Yaacob Latif, Cheras, 56000 Kuala Lumpur, Malaysia

**Keywords:** Miller Fisher syndrome, *Mycoplasma pneumoniae*, Molecular mimicry, GQ1b ganglioside, Antibodies

## Abstract

**Background:**

Neurologic impediments occur in only 0.1% of *Mycoplasma pneumoniae* infections. Although direct intracerebral infection can occur in these patients, autoimmune-mediated reactions secondary to molecular mimicry are the most common pathophysiology of such neurological complications. These complications include immune-mediated encephalitis, peripheral neuritis such as Guillain–Barré syndrome, and many others. Miller Fisher syndrome is a one of the variants of Guillain–Barré syndrome that has been rarely linked to *Mycoplasma pneumoniae* infection. It is a condition classically characterized by the triad of ophthalmoplegia, areflexia, and ataxia. Most patients with Miller Fisher syndrome will have positive anti-ganglioside GQ1b antibodies found in their serum, making this autoantibody a very useful serological confirmation parameter. We report a case of a Miller Fisher syndrome in a woman with *Mycoplasma pneumoniae* infection. To the best of the authors’ knowledge, such cases have been only rarely described in literature.

**Case presentation:**

A 35-year-old Chinese woman presented with sudden onset of double vision and ataxia 5 days after fever and mild flu symptoms. Her *Mycoplasma pneumoniae* antigen was positive with 1 over 2560 titer of total mycoplasma antibody and presence of immunoglobulin M antibody, suggesting acute infection, and her nerve conduction study revealed mild sensory axonal polyneuropathy with segmental demyelination. the Miller Fischer syndrome variant of Guillain-Barré syndrome secondary to *Mycoplasma pneumonia* was suspected and later confirmed by presence of serum anti-GQ1b autoantibody. She was treated with intravenous immunoglobulin 0.4 g/kg once daily for 5 days.

**Conclusions:**

The objective of this report is to share a case of an uncommon neurological complication of *Mycoplasma pneumoniae* infection, to increase the level of suspicion among clinicians that Miller Fischer syndrome can occur as an atypical presentation of an atypical pneumonia.

## Introduction

*Mycoplasma pneumoniae* (MP) is an atypical microorganism that commonly causes community-acquired pneumonia. This organism has peculiar properties that not only make it invisible on the usual Gram stain but also nonsusceptible to the broad-spectrum beta-lactam drugs usually applied as first-line antibiotics to treat community-acquired infection [1]. MP causes atypical pneumonia, associated with a list of extrapulmonary manifestations. These include hemolytic anemia, myringitis, Guillain–Barré syndrome and its variant, and many others. Miller Fisher syndrome is one of the rare extrapulmonary manifestations of MP infection [2]. This atypical presentation of an atypical pneumonia is the center of discussion in this case report.

## Case report

A 35-year-old Chinese female with no known medical illness presented with double vision and body imbalances for the past 2 days. She described that the diplopia was of sudden onset, painless, and did not occur on looking in any specific direction. Regarding the body imbalance, she showed a tendency to sway to the right side. On further questioning, she also had history of preceding fever with mild flu symptoms for the past 5 days.

She also complained of numbness and cramping sensation over the hands and feet bilaterally, 1 day before presentation. Otherwise, there was no neck stiffness, no upper and lower limb weakness, no slurring of speech, no dysphagia, no dyspnea, and no history of recent accident or trauma. Upon arrival to the emergency department, she required assistance for ambulation as she has difficulty in maintaining balance. Her vital signs were stable with blood pressure of 124/78 mmHg, pulse rate of 80 beats per minute, regular rhythm and no collapsing character, afebrile, and not tachypneic. On physical examination, she had diplopia over the lateral gaze bilaterally, but otherwise there was no nystagmus, no dysdiadochokinesia, no dysmetria, and no facial weakness, and examination of the rest of the cranial nerves, upper limbs, and lower limbs revealed no abnormal findings. Romberg’s test was negative, but sharpened Romberg’s test was positive. Her abdomen was soft, not tender, and there was no palpable mass or organomegaly. Examination of the cardiorespiratory system revealed no abnormal findings. Initial blood investigation was normal (Table 1).

**Table 1:** Initial blood investigation taken in the hospital

Blood parameter	Result	Normal range
Hemoglobin	11.2 g/dL	12–18 g/dL
Platelets	266 × 10^9^/L	150–400 × 10^9^/L
White blood cells	5.2 × 10^9^/L	4.0–11.0 × 10^9^/L
Albumin	43 g/L	35–50 g/L
Alkaline phosphatase	78 U/L	50–150 U/L
Alanine transaminase	23 U/L	5–35 U/L
Total bilirubin	6.6 μmol/L	0–13 μmol/L
Creatinine	56.8 μmol/L	60–120 μmol/L
Sodium	135 mmol/L	135–150 mmol/L
Potassium	4.6 mmol/L	3.5–5.0 mmol/L
Urea	6.1 mmol/L	1.7–8.0 mmol/L
Corrected calcium	2.35	2.15–2.55 mmol/L
Magnesium	0.88 mmol/L	0.66–1.07 mmol/L
Phosphate	1.06	0.75–1.50
Serum lactate	1.0 mmol/L	0.5–2.0 mmol/L
Serum creatine kinase	33 U/L	30–200 U/L

Blood investigations taken upon arrival to the hospital showed normal cell counts. There was no electrolyte abnormality, and the renal profile and liver function were also normal.

**Fig. 1:**
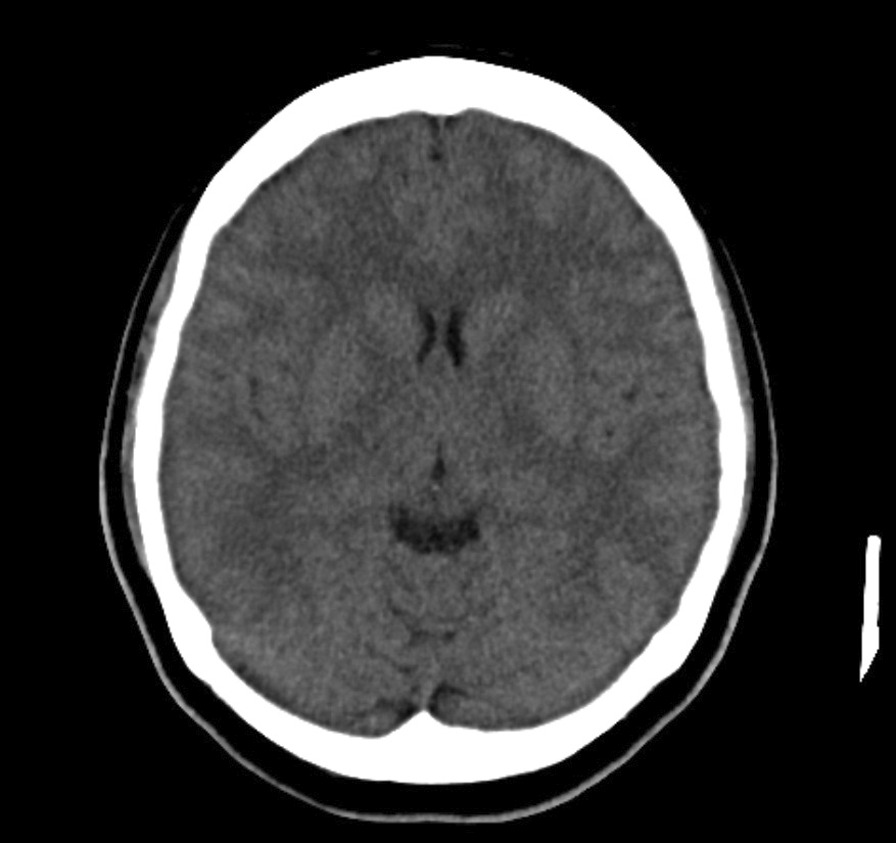
Computed tomographic imaging of brain on admission, showing no intracranial bleeding or space-occupying lesion

**Fig. 2:**
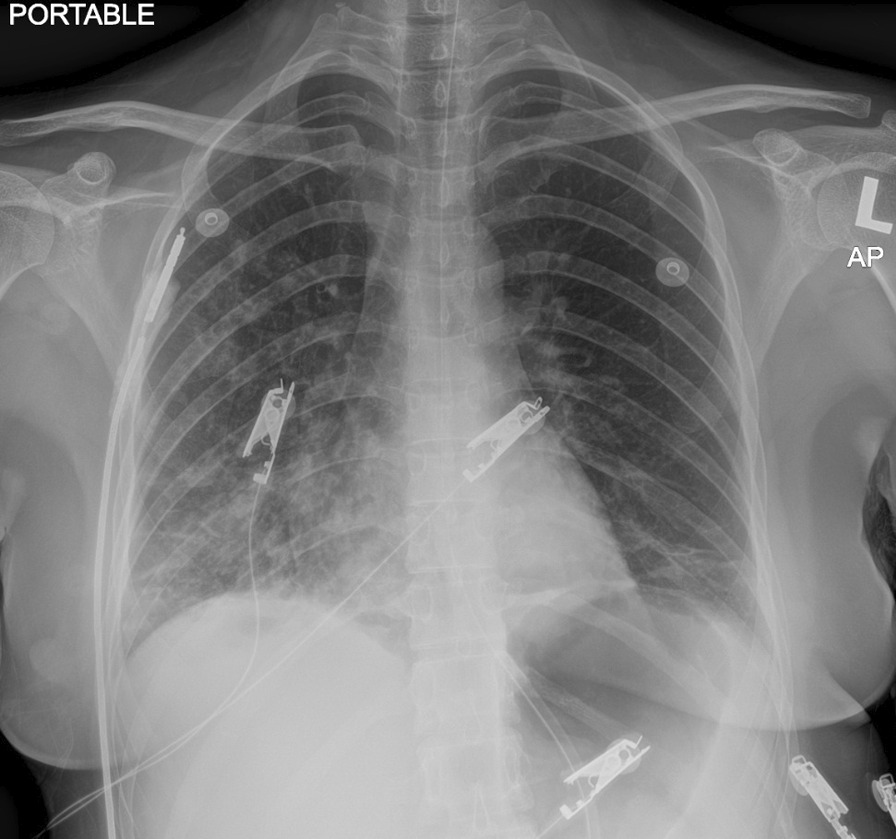
Radiographic imaging of chest taken on second day of admission when the patient developed shortness of breath, showing heterogeneous opacity over the lower right zone

Computed tomographic scan of the brain revealed no intracranial bleeding and no space-occupying lesion (Fig. 1). Nerve conduction study revealed mild sensory axonal polyneuropathy with segmental demyelination. H reflex was absent bilaterally. Lumbar puncture for cerebrospinal fluid analysis was done and showed normal cell counts and biochemistry profile and no abnormal cells, and was negative for bacterial culture, multiple viral antibody panels, and cryptococcal antigen test. In view of the presence of ataxia and diplopia, the Miller Fischer syndrome variant of Guillain–Barré syndrome was suspected and later confirmed with the presence of serum anti-GQ1b autoantibody. Intravenous immunoglobulin (IVIg) 0.4 gm/kg once daily was started and planned to complete for 5 days. On the second day of admission, she developed worsening cough and shortness of breath. Radiographic imaging of the chest revealed homogeneous opacity over the lower right zone (Fig. 2). She was initially started on intravenous (IV) co-amoxiclav 1.2 g three times daily, and was escalated to IV piperacillin–tazobactam 4.5 gm three times daily after 2 days after she failed to show any improvement. As atypical pneumonia was suspected by the treating physician in view of the associated neurological symptoms, *Mycoplasma pneumoniae* antigen was taken and came back positive with 1 over 2560 titer of total mycoplasma antibody and presence of IgM antibody, suggestive of an acute infection. She was started with macrolide antibiotic (oral azithromycin 500 mg once daily for 5 days) on top of the previous IV antibiotic and IVIg treatment. She was also subjected to inpatient physiotherapy for a few days, then discharged well.

## Discussion

*Mycoplasma pneumoniae* (MP) is a common respiratory pathogen. This short, rod-shaped bacterium lacking a cell wall commonly causes respiratory infections such as bronchitis and pneumonia. It is often considered to be an atypical organism as it is not visible on the usual Gram stain and cannot be cultured using standard methods [1]. This organism is also not susceptible to the broad-spectrum beta-lactam antibiotics normally used as first-line treatment for community-acquired pneumonia. MP is one of the causative organisms of “atypical” bacterial pneumonia because the respiratory manifestations are predominated by a complex of constitutional symptoms such as low-grade fever, headache, and malaise, usually associated with extrapulmonary manifestations such as hemolytic anemia, myocarditis, myringitis, encephalitis, and many others that can occur alongside or independent of the respiratory symptoms [2]. Miller Fisher syndrome (MFS) is one of the rare extrapulmonary manifestations of MP infection.

MFS is considered a rare variant of Guillain–Barré syndrome, which is an acute idiopathic, immune-mediated inflammatory polyradiculopathy. The pathophysiology of this condition is still not well understood, but autoantibody-mediated neuritis, a condition in which the immune system attacks the nerves as a result of molecular mimicry that can be triggered by various agents, is a possibility. Among the common pathogens are *Campylobacter jejuni*, *Haemophilus influenzae*, and cytomegalovirus. However, MP is a rare pathogen also found to be associated with MFS. MFS classically presents with a triad of ataxia, ophthalmoplegia, and areflexia. However, the clinical manifestations of MFS can vary widely. The less common presentations include limb dysesthesia, ptosis, facial and bulbar palsies, mild muscle weakness, and urinary incontinence [3]. MFS is two times more common in men and can affect people of all ages, with median age of onset in the fifth decade [4].

The diagnosis of MFS is made clinically. However, serological confirmation with the presence of Anti-GQ1b antibodies is often made to support the diagnosis. This antibody acts on GQ1b, which is a ganglioside found abundantly in the paranodal region of the extramedullary portion of the oculomotor, abducens, and trochlear nerves. These areas are usually those most affected by self-reactive Anti-GQ1b antibodies, blocking release of acetylcholine from the motor nerve ending [4]. Although this antibody is not unique to MFS, it can help support the diagnosis in cases of uncertainty. Moreover, it can also determine the severity of the disease, as the level relates to the disease activity.

MFS is a self-limiting condition, and gradual improvement marks its recovery period and often the resolution of symptoms. Rarely, serious complications such as cardiac arrhythmia or respiratory failure have been reported [5]. Ataxia and ophthalmoplegia typically resolve within 1–3 months after onset, and near-complete recovery is expected within 6 months [5]. Though areflexia may persist, it is not associated with functional disability. Although MFS follows a self-limiting course, immunomodulatory therapies including intravenous immune globulin and plasmapheresis have been used to hasten disease recovery and perhaps decrease the likelihood of progression to more severe conditions such as GBS [6]. Based on the observational data available, complete resolution of ataxia in 1 month and resolution of ophthalmoplegia within 3 months would be acceptable outcome measures [5].

In this report, our patient presented initially with ataxia and diplopia, suggestive of Miller Fischer syndrome that was later confirmed with the presence of anti-GQ1b antibody in serum. She was then treated with IVIg 0.4 g/kg once daily for a total of 5 days. However, she developed worsening respiratory symptoms later on during inpatient immunoglobulin therapy. She did not respond to first-line beta-lactam antibiotics and was investigated for atypical pneumonia, which was later confirmed with the presence of *Mycoplasma pneumoniae* antibody titer in serum and clinically response to macrolide antibiotic. She recovered well and is currently under regular outpatient clinic and physiotherapy follow-up.

## Conclusion

The aim of this case report is to increase the level of suspicion among clinicians regarding the possibility of an association between Miller Fischer syndrome and *Mycoplasma pneumoniae* infection, which should be considered in any cases of pneumonia that develop neurological manifestations such as ophthalmoplegia and ataxia.

## Data Availability

Not applicable.
